# Silencing of the Transmembrane Transporter (*swnT*) Gene of the Fungus *Slafractonia leguminicola* Results in a Reduction of Mycotoxin Transport

**DOI:** 10.3390/jof9030370

**Published:** 2023-03-18

**Authors:** Sumanjari Das, Dale R. Gardner, Marwa Neyaz, Allen B. Charleston, Daniel Cook, Rebecca Creamer

**Affiliations:** 1 Department of Biology, New Mexico State University, Las Cruces, NM 88003, USA; 2 USDA ARS Poisonous Plant Research Laboratory, 1150 E 1400 N, Logan, UT 84321, USA; 3 Plant and Environmental Science, New Mexico State University, Las Cruces, NM 88003, USA; 4 Entomology, Plant Pathology, and Weed Science, New Mexico State University, Las Cruces, NM 88003, USA

**Keywords:** swainsonine, slaframine, transporter, pathogenesis, gene silencing, locoism, *Slafractonia leguminicola*

## Abstract

*Slafractonia leguminicola* infects red clover and other legumes, causing black patch disease. This pathogenic fungus also produces two mycotoxins, slaframine and swainsonine, that are toxic to livestock grazing on clover hay or pasture infested with *S. leguminicola*. Swainsonine toxicosis causes locoism, while slaframine causes slobbers syndrome. The mechanism of toxin secretion by *S. leguminicola* is poorly understood. The aim of this research was to investigate the role of a putative transmembrane transporter, SwnT*,* in mycotoxin transport. The *swnT* gene was silenced by RNA interference using the silencing vector Psilent1, which included inverted repeat transgenes of *swnT*. This resulted in a significant reduction of *swnT* transcript levels compared with the controls. Silencing caused a decline in the active efflux of toxins from the mycelia to the media, as shown by LC–MS analysis. Transformants in which *swnT* was silenced showed higher concentrations of both toxins in the mycelia compared with the concentrations in the media. These transformants exhibited a visibly distinct phenotype with much thicker and shorter mycelia than in the wild type. These transformants were also unable to infect detached clover leaves, unlike the controls, suggesting that SwnT function may play an important role in pathogenesis in addition to mycotoxin transport. This research demonstrates the importance of this transporter to the secretion of mycotoxins for this phytopathogenic fungus.

## 1. Introduction

**Figure 1:**
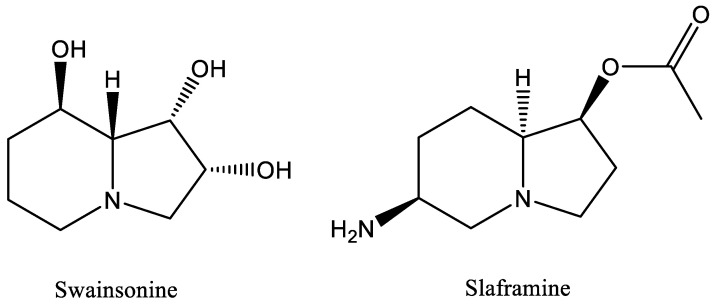
Chemical structure of swainsonine and slaframine, two mycotoxins produced by *Slafractonia leguminicola*.

*Slafractonia leguminicola*, formerly known as *Rhizoctonia leguminicola* [1], infects red clover plants (*Trifolium pratense*) and other legumes such as soybean, kudzu, and cowpea, causing black patch disease of the plants [2]. The fungal pathogen *Slafractonia leguminicola* produces two toxins, slaframine [3] and swainsonine [4] (Figure 1), structurally similar indolizidine alkaloids that are harmful to livestock that consume forages infected with the fungus. The mycotoxin slaframine, 1-acetoxy-6-aminooctahydroindolizine, is responsible for slobbers syndrome [4]. Symptoms of slobbers include excessive salivation, diarrhea, frequent urination, loss of appetite, and even death in severe cases [3]. Slaframine must be converted into an active form to have any physiological effect. The active form is a ketoamine, which is metabolized in the liver [5,6]. Notably, *S. leguminicola* is the only organism reported to produce slaframine.

The other mycotoxin, swainsonine, 1,2,8-trihydroxyoctahydroindolizine, is a cytotoxic alkaloid that inhibits lysosomal α-mannosidase and the golgi mannosidase II, leading to a lysosomal storage disease [7,8]. Swainsonine is toxic to both livestock and wildlife [9]. Swainsonine poisoning is a serious problem in the western United States, causing locoism in cattle, sheep, and horses [9,10,11]. Locoism symptoms include behavioral depression, a staggering walk, lack of muscle coordination, difficulty in eating or drinking, infertility, abortion, and congestive heart failure [9,11]. Swainsonine is also produced by a diverse group of fungi including the *Alternaria* sect. *Undifilum* species, which are seed-transmitted endophytes of *Astragalus* species, *Oxytropis* species and *Swainsona canescens* (Darling Pea), a seed-transmitted symbiont of *Ipomoea carnea* (morning glory family), the insect pathogen *Metarhizium robertsii*, and several species of dermatophytes [12,13].

Recently, an orthologous gene cluster, “*SWN*” (swainsonine), was characterized in four orders of fungi: Hypocreales, Chaetothyriales, Onygenales, Pleosporales [13], and more recently in several other fungal orders [14]. The *SWN* cluster consists of seven genes that encode functional proteins: *swnK,* encoding a hybrid nonribosomal peptide synthetase (NRPS) polyketide synthase (PKS); *swnH1* and *swnH2*, encoding nonheme dioxygenases; *swnR* and *swnN*, encoding reductases; *swnT*, encoding a transmembrane transporter; and *swnA*, which encodes an aromatic amino transferase [13]. Inactivation of *swnK* in *Metarhizium robertsii* resulted in no detectable swainsonine, demonstrating that this gene is required for synthesis of the toxin [13]. Subsequent research by Luo and colleagues investigated the function of the other *SWN* genes in *M. robertsii* [15]. Notably, two *swnK* paralogs with the SwnK domain structure were identified in *S. leguminicola* [13]. Hypothetically, one of the SwnK paralogs may be involved in the biosynthesis of slaframine, and the other in the biosynthesis of a related alkaloid that is yet to be identified. 

Given the structural similarity of swainsonine and slaframine, and since they may share a common biosynthetic pathway [6], it is reasonable to suspect that some genes may be shared between the biosynthetic pathways of each respective alkaloid. SwnT is predicted to be a transmembrane transporter by bioinformatic analyses [13], and has been found among the swainsonine-producing Onygenales, Hypocreales, and one member of the Pleosporales [14]. The role of SwnT has not been investigated in *S. leguminicola*. This research aimed to determine the specific function of the *swnT* gene in *S. leguminicola* and determine any role it may play in swainsonine and slaframine transport, as they are structurally similar indolizidines. The effect of RNA interference (RNAi)-mediated silencing of the *swnT* gene on toxin secretion was tested. Unexpectedly*,* silencing *swnT* also influenced the morphology and the pathogenicity of the fungus. 

## 2. Materials and Methods

### 2.1. Fungal Strains and Culture Conditions

*Slafractonia leguminicola* (strain: RL-4038, ATCC 26280) was used as the wild-type (WT) strain. PDA (potato dextrose agar) or PDB (potato dextrose broth) were used for routine culture of the fungus at 25 °C. Stock cultures of the isolate were maintained on potato dextrose agar (PDA) slants at 4 °C.

### 2.2. Plant Materials

Red clover plants (*Trifolium pratense)* were grown from seed (Source: The Dirty Gardener, Tacoma, WA, USA) and managed routinely in the greenhouse. The plants were approximately 15 cm tall and were flowering when used for inoculations.

### 2.3. Pathogen Inoculations

Fungal cultures were propagated at room temperature on PDA medium. Detached leaves from approximately 30-day-old red clover plants were inoculated in petri dishes containing wet filter paper. For the inoculation of the leaves, a 4 mm plug of 4- to 5-day-old culture was used and was maintained in the growth chamber at room temperature for 9 days. 

### 2.4. SwnT Protein Analyses

SwnT and SwnT-like protein sequences of *Slafractonia leguminicola*, *Fusarium vanettenii*, *Fusarium oxysporum*, *Fusarium fujikuroi*, *Colletotrichum orchidophilum*, *Metarhizium robertsii*, *Metarhizium brunneum*, *Microsporum canis*, *Blastomyces gilchristii*, *Pochonia chlamydosporia*, *Nannizzia gypsea*, *Cyberlindnera jadinii*, *Komagataella phaffii*, *Histoplasma capsulatum*, and *Diplodia corticola* were obtained from the protein blast program of NCBI. Sequences of each protein were added to fasta files and compared with Geneious Prime 2023. The sequences were grouped in a multiple alignment and aligned with MUSCLE alignment (the standard muscle algorithm). The alignment of the 15 sequences produced was selected to build the tree using PAUP* plugin following the maximum parsimony algorithm, heuristic tree search strategy, and bootstrapping (fixed seeds 100, 1000 replications, and FastStep search type). *Cyberlindnera jadinii* was used as the outgroup. 

### 2.5. Generation of Silencing Construct pSilent-swnT

The silencing vector, pSilent-1 [16], was engineered for construction of the RNAi cassette. The pSilent-1 vector carries the *Aspergillus nidulans trpC* gene promoter (P*_trpC_*), intron 2 of a cutinase (*CUT*) gene from *Magnaporthe oryzae* and the *trpC* terminator (T*_trpC_*) for expression of the hairpin cassette, and a hygromycin resistance gene (*hph*) for selection of the transformants. The promoter used for the RNA hairpin formation and for the expression of *hph* was P*_trpC_* [16].

The pSilent-*swnT* was constructed through a two-step process [16,17,18]. Based on the sequence of *S. leguminicola swnT* (KY365742.1), PCR primers (Table 1) were designed to amplify 496 bp fragments of *swnT* to make the inverted repeats in pSilent-1. The *swnT* PCR products were digested by *Xho*I and *Hin*dIII, or *Bgl*II and *Sph*I for cloning in the inverted orientation in pSilent-1, resulting in pSilenT-*swnT* [16,17].

### 2.6. Protoplast Preparation

The protoplasts of *S. leguminicola* were prepared using the methods described by Churchill [19], with some alterations. Mycelia were grown on potato dextrose agar (PDA) for 24 h at 25 C. About 2–4 g of mycelium was homogenized in distilled water and transferred to 50 mL PDB. The fungal cultures were incubated in a shaker at 25 C for 48 h [18]. Following incubation, the hyphal suspension was harvested on sterile Miracloth (Millipore-Sigma) and washed twice with 0.6 M MgSO_4_. The mycelia were collected and resuspended in 25 mL of filter-sterilized digestion buffer prepared in the following way: 5% lysing enzymes (Sigma, L1412), β-glucuronidase, 0.2 mL/g of mycelium (Sigma G-7770), and bovine serum albumin were added to the osmotic medium (1.2 M MgSO_4_, 10 mM sodium phosphate, pH 5.8). The fungal suspension was incubated at room temperature (25 C) and was shaken at 90 rpm for 7–8 h. 

About 10 mL protoplast suspension was collected in a 50 mL polypropylene tube and gently overlaid with a cold trapping buffer (0.4 M Sorbitol in 0.1 M Tris–HCl, pH 7.0) and centrifuged at 4700 rpm for 35 min at 4 C. About 4–5 mL of the protoplasts at the interface were collected in a tube (on ice) and diluted with 2 volumes of 1 M sorbitol. The protoplasts were then pelleted at 7000 rpm for 6 min at 4 C. The pellets were then suspended in 5 mL of STC buffer (1 M sorbitol in 100 mM Tris–HCl, pH 8.0, 0.1 M CaCl_2_) and centrifuged at 7000 rpm for 10 min at 4 C. Supernatants were removed and the collected protoplast pellets were suspended in STC buffer and kept on ice for the transformation experiments. 

### 2.7. Chemical Transformation of Slafractonia Leguminicola Protoplast

The WT *S. leguminicola* strain was transformed with uncut Psilent-*swnT* by a PEG-mediated protoplast method [19,20]. Transformants were selected on potato dextrose agar (PDA) containing hygromycin B [17,18]. 

Freshly prepared protoplasts (100 μL) were transferred to a precooled 15 mL Falcon tube on ice. About 8 μg of *swnT* plasmid DNA diluted in 10 μL of Tris–EDTA (TE) buffer was added to the protoplast and incubated on ice for 30 min. Ten microliters of Tris–EDTA (TE) buffer without DNA was added to the protoplasts as a control. Protoplasts with only pSilent-1 plasmid (empty vector) were transformed as an additional control.

One milliliter of PTC buffer (PTC buffer: 40% Polyethylene Glycol, 4000, 0.1 M Tris–HCl, pH 8.0, and 0.1 M CaCl_2_) was added to each tube and incubated at room temperature for 25 min. About 200 μL of the transformation mixture was pipetted onto petri dishes. Thereafter,12.5 mL of regeneration medium (1 M of sucrose, 0.001 *w/v* of yeast extract, 0.001 *w/v* of casein hydrolysate, and 0.016% of Bacto agar) was added to each plate and swirled to mix. The plates were incubated for 24 h at 25 C and then overlaid with a 12.5 mL regeneration medium containing 70 μg/mL hygromycin B (Hyg) [17,18,19]. The plates were further incubated at 25 C for 7–10 days until hyphae were detected growing through the top layer. The putative transformants were transferred to PDA-Hyg plates. The hyphae from TE control plates and empty vector were also transferred to PDA without antibiotics to compare phenotypes to that of the putative transformants. To confirm stability, colonies from PDA-Hyg were transferred to non-selective PDA plates 3 times (3 fungal passages) followed by transfer to a PDA plate with 70 μg/mL hygromycin B [16]. The colonies that could propagate at least 2–5 cm on the PDA plates in a week were considered stable transformants [20,21,22].

### 2.8. Screening of swnT Transformants

To confirm that transformants contained the silencing vector, the hygromycin B-resistant cassette (300 bp) was amplified by PCR using primers cdH2/cdH3 (Table 2) and the PCR products were sequenced. Agarose gel electrophoresis showed that all the transformants contained the *hygB* transgene, whereas the WT control had no product.

### 2.9. Preparation of Total RNA and cDNA

On day 3 post-transformation, total RNA was extracted from six *swnT* transformants and the controls—three biological replicates for each sample—using TRIzol reagent (Thermo Fisher Scientific, Waltham, MA, USA # 15596026). cDNA for subsequent qPCR analysis was prepared from 1 μg of the isolated total RNA using SuperScript™ IV First-Strand Synthesis System (Thermo Fisher Scientific # 18091150) following the manufacturer’s instruction.

### 2.10. Reverse-Transcription-Quantitative PCR (RT-qPCR)

Reverse transcription quantitative PCR of different transcripts was performed by qPCR on an CFX Connect Real-Time PCR system (Bio-Rad) using iTaq Universal SYBR Green Supermix (# 1725121 Bio-Rad) following the manufacturer’s protocol. The expression of each gene of interest (Ct value) was normalized against RDN5.8 mRNA. The primers used were SSL1/SSL2 for *swnT* and RDN5.8F/RDN5.8 R for RDN5.8 (Table 2). The reaction mixtures (20 μL) contained 10 μL of iTaq Universal SYBR Green Supermix. The PCR conditions were 95 C for 30 s, followed by 40 cycles of 95 C for 5 s, and 60 C for 30 s, followed by a melting curve analysis. RT-qPCR was conducted in triplicate for each sample and the data were analyzed using the 2^−ΔΔCT^ relative quantification method. The differential expression pattern of *swnT* was compared using an unpaired Student’s *t*-test.

### 2.11. Chemical Analysis of Swainsonine and Slaframine

#### 2.11.1. Extraction

Six selected *swnT* mutants, the WT, and the WT transformed with the empty vector pSilent-1 were grown on PDA plates for four days at 28 °C. The mycelial mass of each culture was dried in the oven for 3 h at 80 °C. A measured mass of dry mycelia from each culture was weighed and extracted for swainsonine and slaframine. A measured volume of 95% ethanol was added, and the samples extracted for 16 h with mechanical rotation. A measured mass of dried agar was placed into a 7 mL screw cap vial, a measured volume of 95% ethanol added, and the samples were extracted for 4 h with mechanical rotation. Aliquots of the extracts (0.500 mL from fungal extracts and 0.100 mL from agar extracts) were diluted with an equal volume of deionized water into autosampler vials.

The swainsonine (0.50 mg/mL) and slaframine (0.30 mg/mL slaframine; 1.0 mg/mL as slaframine dipicrate) were diluted (0.020 mL swainsonine solution and 0.033 mL slaframine solution) into 0.950 mL of 50% methanol to give a 10 ppm standard solution. A 0.200 mL aliquot was added to 1.80 mL of 50% methanol to give a 1000 ng/mL solution that was serially diluted to give standards at 1000, 500, 250, 125, 62.5, 31.2, 15.6, 7.8, 3.9, and 1.95 ng/mL. 

#### 2.11.2. HPLC-HRMS Analysis 

The standards and sample extracts were analyzed by high-performance liquid chromatography coupled with high-resolution mass spectrometry (HPLC–HRMS) using the following instrumentation. A Q-Exactive quadrupole/orbitrap high-resolution mass spectrometer (Thermo Fisher Scientific) equipped with a heated electrospray ion source (HEIS) and coupled to a UltiMate 3000 HPLC (Thermo Fisher Scientific) was used for the analyses. The samples were chromatographed using a Hypercarb column (100 × 2.1 mm, 5 µm: Thermo Fisher Scientific) and a binary mixed solvent system of 20 mM ammonium acetate (A) and methanol (B) flowing at 0.400 mL/min. The gradient mixture was programed as follows: 5% B (0–1 min), 5–70% B (1–5 min), 70% B (5–8 min), 70–75% B (8–10 min), and 5% B (10–15 min). The flow from the column was connected directly to a HEIS of the mass spectrometer and calibrated as per the manufacturer’s instructions and with a scan range 100–800 Da (positive ion), resolution 35,000, micro scans 1, sheath gas flow 35, auxiliary gas flow 10, spray voltage 4 kV, capillary temperature 320 C, S lens RF field 55, and auxiliary gas temperature 300 °C. Detection and peak areas were obtained from the area under the curve of reconstructed ion chromatograms (RIC) of selected ions at *m/z* = 174.1122 ± 5 ppm (swainsonine) and *m/z* = 199.1437 ± 5 ppm (slaframine). The swainsonine and slaframine concentrations were compared using a chi square test.

Pearson’s correlation coefficient was calculated between the percentage of *swnT* gene expression relative to the WT, and the ratio of swainsonine or slaframine concentration in the mycelia to the agar using GraphPad Prism. The correlation coefficient, denoted by r, is a measure of the strength of the linear relationship between the two variables on the x-axis and y-axis, respectively.

### 2.12. Inoculation of the Detached Leaves with the swnT T5 Transformant and S. leguminicola

Detached red clover leaves were inoculated with *swnT T5* transformant and WT five-day-old cultures of *S. leguminicola.* The leaves were placed on a petri plate with wet filter paper and were inoculated with a 4 mm plug of both the *swnT T5* transformant and the WT. The appearance of lesions was observed at day 4 and day 20, and photographed under a differential interference contrast (DIC) microscope.

## 3. Results

### 3.1. SwnT Protein

**Figure 2:**
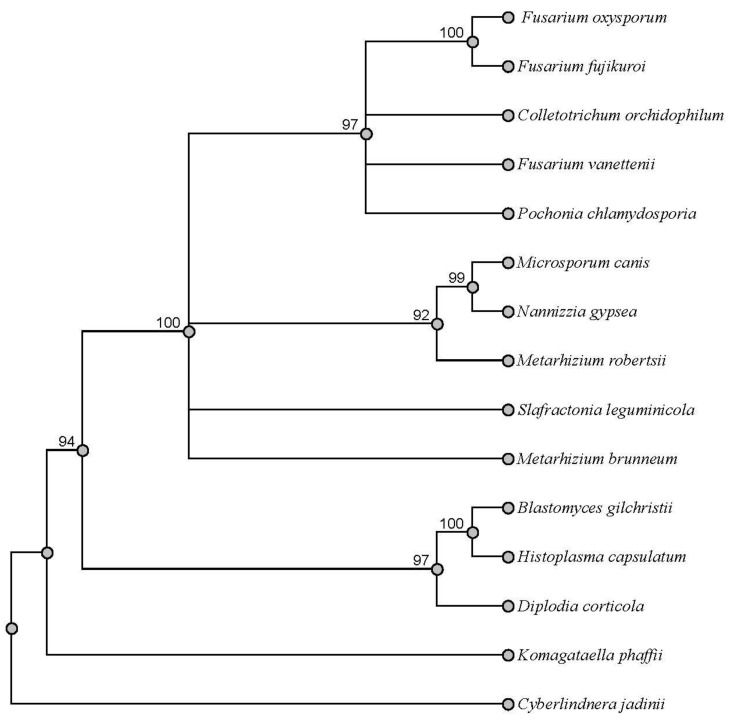
Phylogenetic comparison of SwnT and other related proteins. Protein sequences of each organism were compared with Geneious Prime 2023. The sequences were aligned with MUSCLE alignment and the tree built using PAUP* plugin following the maximum parsimony algorithm with 1000 replications. *Cyberlindnera jadinii* was used as the outgroup.

The phylogenetic tree shows that SwnT from *S. leguminicola* is most closely related to transporters from two groups of fungi, a clade of Ascomycete fungi, representing a subgroup including *Metarhizium* and *Microsporum*, whose members contain the *SWN* cluster and some of which have been reported to produce swainsonine (13); and another subgroup, including species of *Fusarium* and *Colletotrichum*, whose members have not been reported to produce swainsonine (Figure 2). BLASTp analysis using the full-length protein sequence of SwnT showed that the closest hits shared less than 70% identity with the proteins predicted to be amino acid/polyamine transporters from the genera of *Fusarium* and *Colletotrichum* in the phylogenetic tree. 

### 3.2. Selection, Expression, and Phenotype of the swnT-silencing Transformants

**Figure 3:**
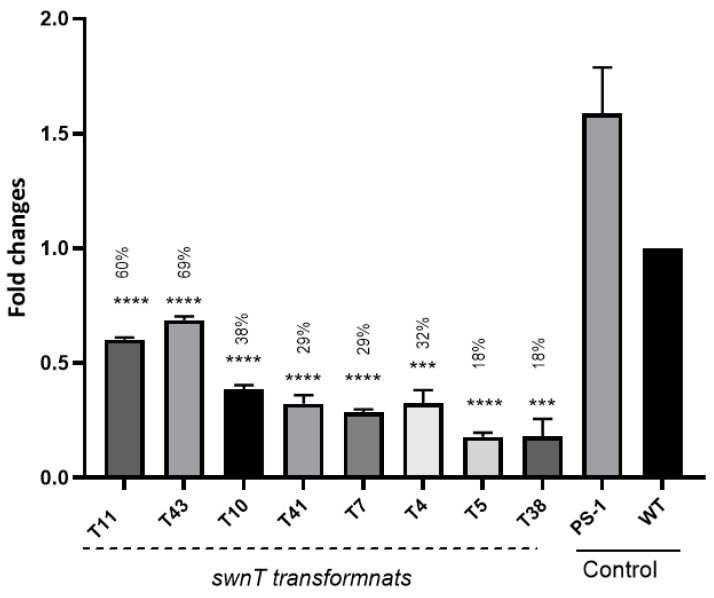
Reverse transcription quantitative PCR (RT-qPCR) analysis of total RNA of WT and *swnT-*silencing transformants. Target *swnT* gene expression was normalized to the expression pattern of fungal internal control gene RDN5.8. WT: wild-type *S. leguminicola*, PS-1: S*. leguminicola* transformed with empty vector (pSilent-1), pSilent-*swnT* transformants (T10, T41, T7, T5, T11, T43, T38 & T4) growing on PDA for four days after transformation with the silencing construct pSilent-*swnT*. Fold change is normalized to WT, and percentages represent percentage of *swnT* expression relative to WT. Error bars represent the ± SEM (*n* = 3), *p* values are calculated using unpaired Student’s t test compared with WT ( *** *p* < 0.001, **** *p* < 0.0001, ns = not significant *p* > 0.05).

The RNA-interference expression cassette for *swnT* (pSilent-*swnT*) was transformed into *S. leguminicola* protoplasts. Within 7–10 days, colonies appeared on the hygromycin B plates. Eight hygromycin-resistant transformants were randomly selected. The presence of pSilent-*swnT* was confirmed in all eight randomly selected transformants, and the colonies were subsequently transferred to PDA for further evaluation. The qRT-PCR results demonstrated that the *swnT* gene was expressed in the WT strain and the WT transformed with the empty vector pSilent-1 at similar levels. The mRNA level of *swnT* was significantly decreased in all the transformants, ranging from approximately 31% to 82% reduction compared with that of the controls (Figure 3). The transformants T5 and T38 showed the highest level of silencing. 

**Figure 4:**
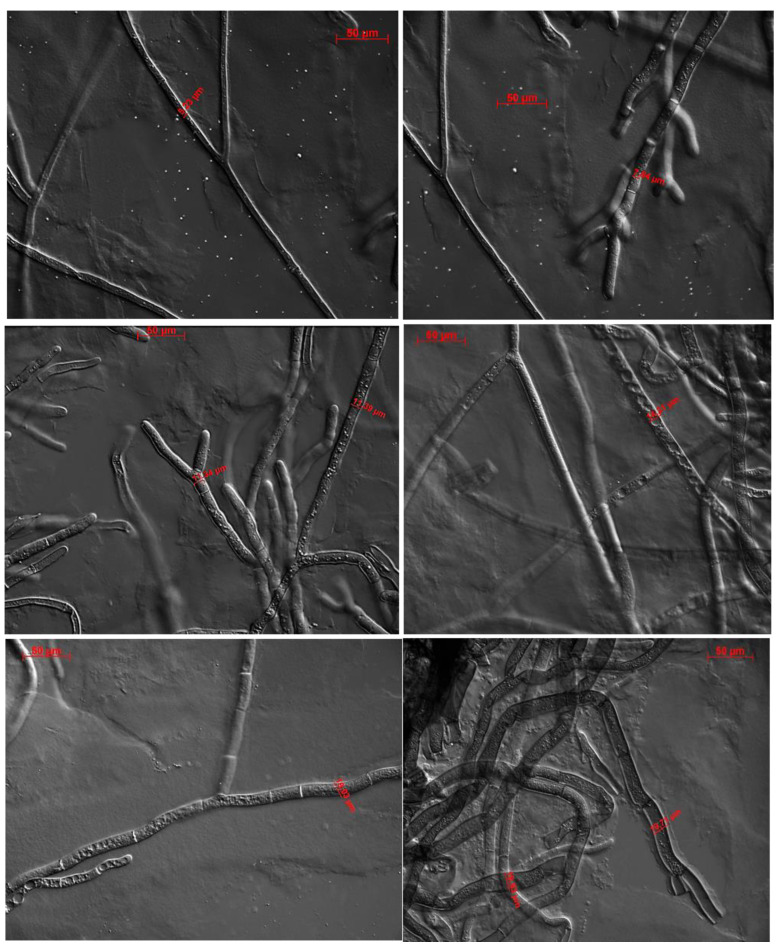
Morphological phenotypes of hyphae of the *swnT* transformants and WT *S. leguminicola.* Differential interference contrast (DIC) micrographs of *S. leguminicola* hyphae grown at 26 C for 72 h. WT hyphae (upper panel) showing hyphal width 5.23 μm to 7.84 μm. *swnT* transformed T10 (middle panel) hyphal width 11.34 μm to 14.21 μm and pSilent-*swnT* transformed T5 (lower panels) hyphal width 19.03 μm to 19.83 μm. Bar, 50 μm.

Transformants with about 82% silencing of *swnT* (T5 and T38) showed very poor mycelial growth and a slower growth rate than the WT. Some transformants with higher levels of *swnT* silencing were not able to survive, while the transformants with about 60% silencing of *swnT* appeared to have a normal growth pattern similar to that of the WT. Differences were observed in hyphal structure between the transformants and the control (Figure 4).The *swnT-*silenced transformants showed wide, stunted hyphae, whereas the WT showed long, thin hyphae. The width of the hyphae of the *swnT* transformants (T5 and T38 with 82% silencing) ranged from 18.0 μm to 29.5 μm, with an average of 22.2 μm, whereas the width of the hyphae of the WT ranged from 5.2 μm to 8.2 μm, with an average of 7.1 μm. The transformants with a moderate level of silencing (T10, T4 with 62–71% silencing) showed hyphal width ranging from 7.1 μm to 13.2 μm. 

### 3.3. Comparison of Swainsonine and Slaframine Concentrations (Ratio) in Mycelia Versus the Agar

**Figure 5:**
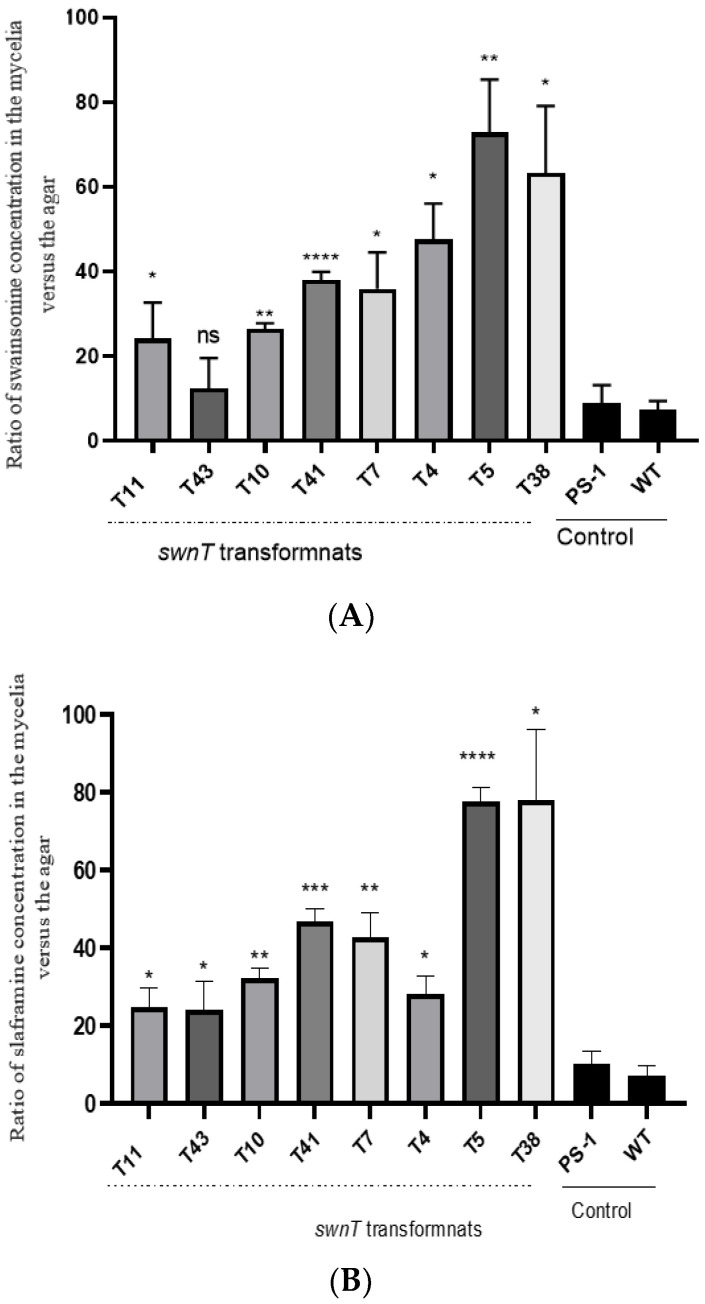
Biochemical analysis of (**A**) swainsonine and (**B**) slaframine (LC–MS) in the pSilent-*swnT* transformants. Data are expressed as ratios of the (**A**) swainsonine and (**B**) slaframine concentrations in the mycelia versus the agar between the pSilent-*swnT* transformants, PS-1 *S. leguminicola* transformed with empty vector (pSilent-1), and WT *S. leguminicola*. Significant differences in the ratios were observed between the transformants and the controls. Transformants T5 and T38 showed the highest ratio compared with the control. Error bars represent the ± SEM (*n* = 3), *p* values are calculated using unpaired Student’s t test compared with WT (* *p* ≤ 0.05, ** *p* < 0.01, *** *p* < 0.001, **** *p* < 0.0001, ns = not significant *p* > 0.05).

**Figure 6:**
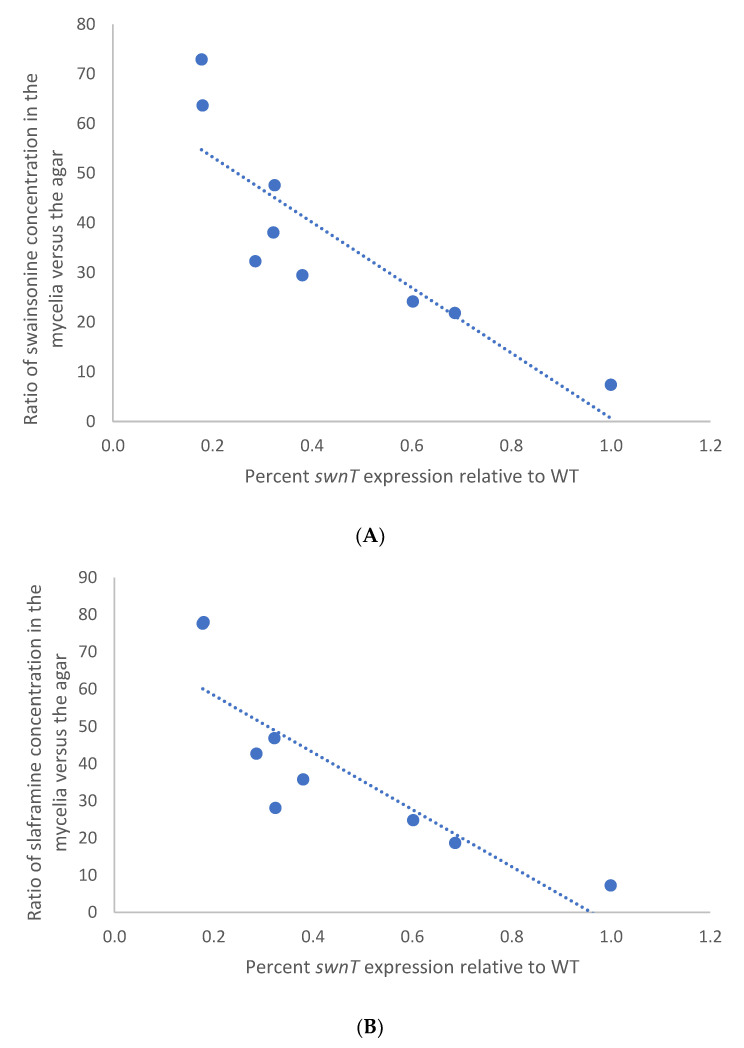
Pearson correlation between percentage of *swnT* expression relative to WT and ratio of (**A**) swainsonine and (**B**) slaframine concentration in the mycelia versus the agar in the pSilent-*swnT* transformants and WT. Correlation coefficient was used to determine the relationship between *swnT* gene expression and the ratio of (**A**) swainsonine (r = −0.86) and (**B**) slaframine (r = −0.85) concentrations in the mycelia versus the agar.

Silencing *swnT* altered the ratio of the swainsonine and slaframine in the mycelia relative to the agar. There was a statistically significant higher ratio of swainsonine and slaframine in the mycelia relative to the agar in the *swnT*-silenced transformants compared with the WT and the WT transformed with the empty vector, pSilent-1. The transformants that showed the greatest amount of silencing (T5 and T38) showed the highest ratio of swainsonine and slaframine in the mycelia versus the media (Figure 5 A,B). The correlation coefficient between the *swnT* gene expression among different transformants and the swainsonine ratio and slaframine ratio was −0.86 and −0.85, respectively, indicating a strong negative correlation in both cases (Figure 6A,B).

### 3.4. Role of swnT in Fungal Pathogenesis

**Figure 7:**
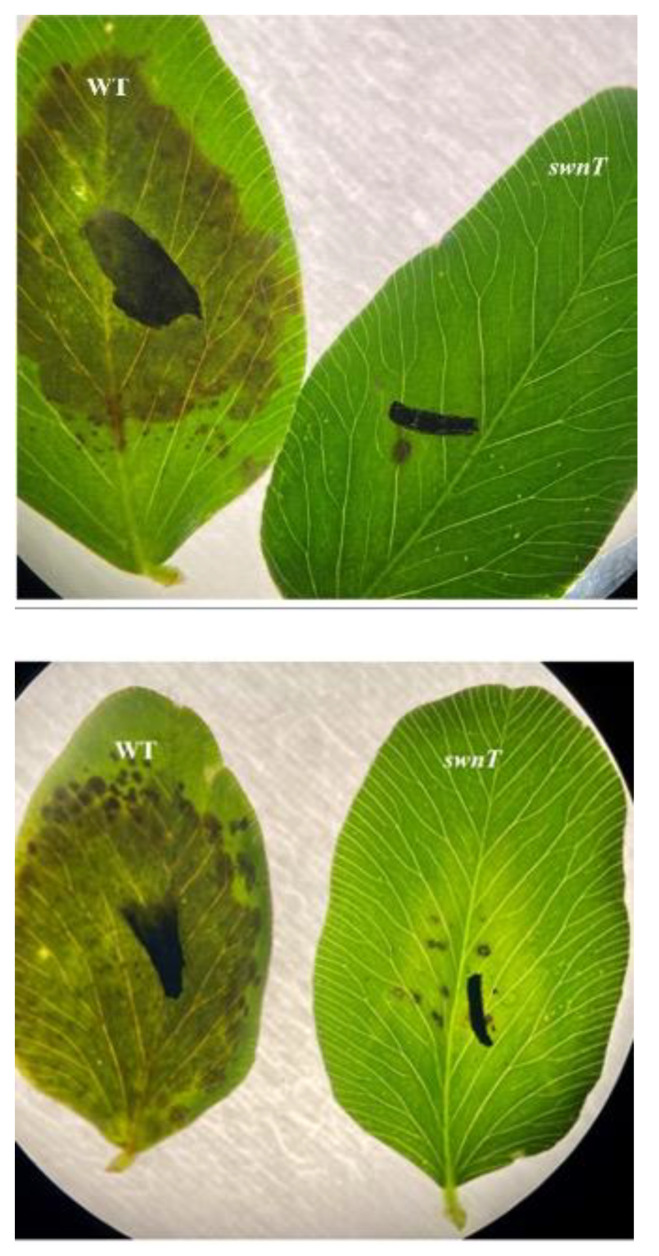
Detached red clover leaves infected with the WT and the T5 pSilent-*swnT* transformants (4 dpi) (black marks on the leaves were drawn on the leaf at time of inoculation for a point of reference). Detached red clover leaves were inoculated with the T5 *swnT* transformant and WT five-day-old cultures of *S. leguminicola.* On day 4 post-inoculation, the leaves inoculated with WT *S. leguminicola* showed black patch lesions covering almost the entire leaves, while the leaves inoculated with T5 pSilent-*swnT* transformants showed very few lesions.

Detached red clover leaves were inoculated with the T5 transformant and the WT five-day-old cultures of *S. leguminicola.* On day 4 post-inoculation, the WT leaves showed black patch lesions covering almost the entire leaves, whereas the leaves inoculated with the T5 *swnT* transformants showed very few lesions (Figure 7). On day 10 post-inoculation, the leaves inoculated with the WT fungus wilted and turned gray or dark brown, while the leaves inoculated with the T5 pSilent-*swnT* transformants were still green and showed limited lesions.

## 4. Discussion

There are limited studies investigating the function of different proteins in the plant pathogen *S. leguminicola*. To date there is a single study, which investigated a gene, *pks1*, that encodes a polyketide synthase involved in the production of melanin [17]. Herein, the function of SwnT was investigated using RNAi-mediated silencing in *S. leguminicola*. We confirmed that *S. leguminicola* produces both swainsonine and slaframine, consistent with previous reports [3,6]. We found that the transport of both swainsonine and slaframine from the mycelia to the media was reduced in the pSilent-*swnT* transformants. Statistically significant differences in the ratio of swainsonine and slaframine concentrations were found in the mycelia versus the media in the *swnT*-silenced transformants compared with the WT. These results suggest that SwnT is likely to play a role in the transport of swainsonine and slaframine in *S. leguminicola*. Silencing *swnT* also resulted in a morphological phenotype: *swnT*-silenced transformants showed poor mycelial growth on plates and had increased widths of hyphae. Notably, WT *S leguminicola* was able to cause plant disease, as has been previously reported [2], unlike the *swnT*-silenced transformants, which showed poor mycelial growth on the red clover leaves and were no longer able to cause plant disease.

In this study, we did not mutate the *swnT* gene, but instead it was silenced. Transformants with about 82% silencing of *swnT* showed very poor mycelial growth and a slower growth rate compared with the WT, and some transformants with higher levels of *swnT* silencing could not survive. The data suggest that silencing *swnT* may have some pleiotropic effects that may influence the overall fitness of *S. leguminicola*, altering its growth and ability to cause plant disease. 

SwnT is similar to proteins that are predicted to be amino acid/polyamine transporters from two other groups of fungi, one representing a subgroup including *Metarhizium* and *Microsporum*, whose members contain the SWN cluster and some of which have been reported to produce swainsonine, and another representing another subgroup including species of *Fusarium* and *Colletotrichum*, whose members have not been reported to produce swainsonine. The role of SwnT-like proteins in these fungi has yet to be investigated, with the exception of *M. robertsii*. In addition, the function of SwnT in *S. leguminicola* may be in addition to an as yet undescribed role that it shares with these other fungi that contain a similar protein. 

The results reported herein differed from the *swnT* deletion data reported in *M. robertsii* [15]. Swainsonine production was reduced in the mycelia and greater in the culture filtrate of the null mutants in *M. robertsii* [15], and no effect on growth or morphology was reported for the *M. robertsii swnT* mutants. The authors concluded that SwnT had no function in the secretion of swainsonine, and suggested that it may instead be involved in the uptake of amino acid nutrient(s) [15]. There are many possible explanations for why the results found for silencing *swnT* in *S. leguminicola* were not the same as for deletion of the gene in *Metarhizium*. The disparity of *swnT* inactivation data between *S. leguminicola* and *M. robertsii* may be due to the fact that *S. leguminicola* is a plant pathogen whereas *M. robertsii* is an entomopathogen. *Slafractonia leguminicola* produces slaframine as well as swainsonine, whereas *M. robertsii* produces swainsonine but not slaframine. 

Lastly, we report a method for the detection and quantitation of slaframine. Since no other fungus has been reported to produce the toxin, the ability to study slaframine in a precise manner is a significant addition to the study of the *S. leguminicola*. In summary, this study provides novel data regarding the potential role of SwnT in the transport of swainsonine and slaframine, and the importance of this protein in the pathogenicity of this important and unique fungus. 

## Figures and Tables

**Table 1:** PCR primers used for *swnT* cloning. Restriction sites are in boldface type.

Primer	Sequence (5′→3′)
SSL5 *Xho*1 (F)	TAGAAG**CTCGAG**TGTCAATGTCTCTGGGTGGC
SSL6 *Hin*dIII (R)	AGTCT**AAGCTT**GAGGTAGAGACAGCCGAGGA
SSL5 *Sph*1 (F)	TAGAAG**GCATGC**TGTCAATGTCTCTGGGTGGC
SSL6 *Bgl*II (R)	AGTCT**AGATCT**GAGGTAGAGACAGCCGAGGA
SSL7 *Xho*1 (F)	TAGAAG**CTCGAG**CGCCACTAGCTTCTTCACCA
SSL8 *Hin*dIII (R)	AGTCT**AAGCTT**GGGAGTTGAAGGCCAGTTCA
SSL7 *Sph*I (F)	TAGAAG**GCATGC**CGCCACTAGCTTCTTCACCA
SSL8 *Bgl*II (R)	AGTCT**AGATCT**GGGAGTTGAAGGCCAGTTCA

F (Forward), R (Reverse).

**Table 2:** Primers used for RT-qPCR of *swnT*.

Primer Name	Sequence (5′→3′)
SSL1 (F)	TCCGGCTTGCTTGTCATCTT
SSL2 (R)	GATTCACGGCTCAGTGTCCA
cdH2 (F)	TCCATACAAGCCAACCACGG
cdH3 (R)	GCGTGATTTCATATGCGCGA
RDN 5.8 (F)	CTTGGTTCTCGCATCGATGA
RDN 5.8 (R)	GGCGCAATGTGCGTTCA

F (Forward), R (Reverse).

## Data Availability

Not applicable.
